# Supplementary material for: Collaborative intelligence in AI: Evaluating the performance of a council of AIs on the USMLE
Source: PLOS Digit Health. 2025 Oct 9;4(10):e0000787. doi: 10.1371/journal.pdig.0000787 (PMC12510544; doi:10.1371/journal.pdig.0000787)
Supplement: S2 File — (ZIP) [file pdig.0000787.s002.zip › S2 Data Raw data files containing unprocessed question inputs and LLM outputs including transcripts of deliberations/STEP3_Question-94-1_Type-MC-NJ.docx]

**Facilitator** **[Round 1]:** A 22-year-old woman comes to the emergency department because of a 5-day history of sore throat and fever. During the past 3 days, she also has noticed a change in her voice and has had increased pain with swallowing. She has been taking acetaminophen since her symptoms began. Medical history is unremarkable and she takes no routine medications. Vital signs are temperature 40.0°C (104.0°F), pulse 130/min, respirations 18/min, and blood pressure 102/66 mm Hg. Pulse oximetry on room air shows an oxygen saturation of 97%. Examination of the oropharynx shows erythema, edema, and anterior displacement of the right side of the soft palate. The uvula is deviated to the left. There is a white exudate on the right tonsil. Trismus is noted. Palpation of the neck discloses enlarged, tender, mobile anterior cervical lymph nodes. In addition to antibiotic therapy, which of the following is the most appropriate management? (A) Incision and drainage (B) Intravenous methylprednisolone therapy (C) Oral nystatin therapy (D) Salt water gargle (E) Tonsillectomy

**AI_B:** RESTATING THE QUESTION: A young woman has been dealing with a sore throat and fever for nearly a week; additionally, for the past couple of days, the woman's voice has changed, and swallowing has caused increased pain. She has taken acetaminophen in an attempt to alleviate her symptoms, which are accompanied by high fever and elevated pulse rate. Examination of her throat reveals redness (erythema), swelling (edema), significant change in the anatomical orientation of the palette (anterior displacement of the right side of the soft palate, with uvula deviated to the left), and visible pus (white exudate) on her right tonsil. In addition to these symptoms, she has difficulty opening her mouth (Trismus), and her anterior cervical lymph nodes are swollen and tender. The question asks what course of management should be applied, beyond antibiotics. PLANNING: The key assumptions that can be made from the presented symptoms are: 1) She has a severe throat infection, possibly tonsillitis with right-side peritonsillar abscess; 2) The displacement of the soft palate and uvula indicates a substantial swelling is present, which could be linked to an abscess formation; 3) The enlarged anterior cervical lymph nodes indicate systemic involvement; 4) The pain upon swallowing (odynophagia) and voice changes (hot potato voice) further support the possibility of a peritonsillar abscess. CLINICAL REASONING: Considering these assumptions, and recalling from our clinical knowledge that peritonsillar abscess (PTA) is often a complication of untreated, or inadequately treated acute tonsillitis, its symptoms precisely correlate with the patient's current condition. PTA is characterized by tonsillar infection that extends into the peritonsillar space. This extension can cause the deviated uvula and soft palate changes seen in this patient. Therefore, antibiotic therapy alone might not be sufficient treatment, and a procedure to drain the abscess could be beneficial to speed up recovery, reduce pain, and prevent complications. CONCLUDING: Given the symptoms, findings, and the reasoning explained above, the most clinically prioritized option would be to proceed with surgical incision and drainage. RESPONSE: Thus, the most appropriate management for this patient, in addition to antibiotic therapy, would be (A) Incision and drainage.

**AI_A:** RESTATING THE QUESTION: The patient, a 22-year-old woman, has come to the emergency department with symptoms of a severe sore throat, fever, change in voice, and increased pain while swallowing. Her symptoms have persisted for days, and acetaminophen has not reduced them. A physical examination shows signs of inflammation in the oropharynx, swollen cervical lymph nodes, and other signs suggestive of a severe throat infection. We are being asked to identify the most appropriate management in addition to antibiotic therapy. The options provided include incision and drainage, intravenous methylprednisolone therapy, oral nystatin therapy, salt water gargle, and tonsillectomy. PLANNING: The symptoms described and physical examination findings suggest that the patient has peritonsillar abscess (PTA), a complication of an untreated, severe bacterial throat infection, often related to streptococcal species. The primary assumption would be to manage not only the infection but also the abscess. However, considering the alternate assumption, mere antibiotic therapy might not suffice as the abscess itself can present a continued infectious focus and a risk of complications like airway obstruction. So, clinical priority should be given to tackling the abscess in addition to the infection. CLINICAL REASONING: Peritonsillar abscess, also known as quinsy, is a collection of infected material in the peritonsillar space. It usually presents with symptoms of severe sore throat, pain on swallowing, change in voice (often termed "hot potato voice"), fever, and trismus due to inflammation of the muscles of mastication. Physical exam may show uvular deviation, edema of the surrounding tissue, and neck lymphadenopathy. While antibiotic therapy is essential to manage the underlying infection, it wouldn't directly deal with the abscess, which can potentially obstruct the airway or contribute to ongoing infection. PTA is essentially a 'walled off' infection, and this compartmentalization may prevent adequate penetration of antibiotics. So drainage is crucial and recommended in these cases. CONCLUDING: Therefore, considering all the information and clinical reasoning, it becomes apparent that tackling the abscess and infection simultaneously would be the best clinical course of action for this patient. RESPONSE: So, the most appropriate management for this patient, in addition to antibiotic therapy, would be (A) Incision and drainage.

**AI_D:** The patient's presentation in the emergency department brings forth multiple symptoms, predominantly related to her throat. Her history of doling with a sore throat, fever, and subsequent alterations in her voice, increased swallowing difficulty, and noticing improvements with acetaminophen, signal towards an infection localized at the throat. The examination findings of erythema, edema, anterior displacement of one aspect of the soft palate, uvular deviation, tonsillar exudate, trismus, and enlarged, tender, mobile anterior cervical lymph nodes collectively provide a strong inclination towards an oropharyngeal infection. On a technical note, trismus refers to difficulty or pain in opening the mouth due to spasms of masticatory muscles. In selecting the most appropriate clinical assumption among the available alternative treatments, the biomechanical situation of soft tissue infection in the throat, its influence on normal physiology, and evaluation based on the infection's severity become crucial. Notably, the analysis should consider the most common oropharyngeal infections, namely pharyngitis, tonsillitis, and peritonsillar abscess. Resorting to clinical reasoning, this certain case represents symptoms potent enough to suggest a peritonsillar abscess, instead of simple pharyngitis or tonsillitis. Noteworthy symptoms include severe sore throat, uvular deviation, trismus, and anterior tonsillar pillar displacement, frequently characteristic of an ongoing peritonsillar abscess. Moreover, it is essential to relieve the abscess considering, firstly, the immediate threat it poses through further spread of the infection, and secondly, to alleviate the intense pain and swallowing difficulties in the patient. Drawing a conclusion from the presented clinical reasoning, incision and drainage appear a suitable resolution in the current scenario. Although antibiotics serve as a crucial element in treatment for combating the infection, local surgical intervention becomes necessary with abscess formation. The purpose is to evacuate collected pus, thus relieving pain, improving swallowing and preventing potential complications, such as airway obstruction, abscess rupture into the deep neck spaces or sepsis. Hence, the response to the query, 'In addition to antibiotic therapy, which of the following is the most appropriate management?', considering the clinical progression chartered, would be (A) Incision and drainage. This step not only supports clearing the infection effectively but also aids manage the symptoms substantially, thus linking with the prime emblem in medical practice, which relates to improving patient's health and alleviating suffering.

**AI_E:** RESTATING THE QUESTION: The scenario describes a 22-year-old woman presenting with a sore throat, fever, changed voice, difficulty swallowing, as well as a significant clinical examination suggesting an infectious process located in the oropharynx. The primary question is regarding the optimal additional intervention to antibiotic treatment, given the possible options of: incision and drainage, intravenous methylprednisolone therapy, oral nystatin therapy, salt water gargle, and tonsillectomy. PLANNING: The key points in this case include the patient's symptoms and the clinical findings, particularly the erythema, edema, and displacement of the soft palate and uvula, as well as the white exudate on the tonsil—all indicating a significant infection. The options given for additional management are all viable strategies for various conditions, which leads to an alternate assumption that the condition to be addressed is not simply a common infection like pharyngitis or tonsillitis. CLINICAL REASONING: One must think about conditions that extend beyond the common throat infection, given the severity and the particular characteristics of this case. In this context, the displacement of the uvula and the signs of an enlarged structure in the oropharyngeal area suggest a unilateral mass, presumably an abscess - specifically, peritonsillar abscess. This condition, often arising as a complication of tonsillitis, is one of the few in which incision and drainage would be appropriate, required even. In contrast, corticosteroids such as methylprednisolone could alleviate symptoms but would not address the core issue. Oral nystatin would be used for a fungal infection, which this is not. Salt water gargles can soothe a sore throat, but they're not adequate for abscess management. Tonsillectomy is a more drastic measure typically reserved for recurrent episodes of tonsillitis or obstructive sleep apnea and would not generally be done in an acute infectious setting. CONCLUDING: Given the specific details described in the exam and the process of elimination, the patient most likely has a peritonsillar abscess, a complication of tonsillitis that often presents with severe sore throat, fever, voice changes (termed 'hot potato voice'), and difficulty swallowing, plus physical findings such as uvula deviation, trismus, and cervical lymphadenopathy. RESPONSE: The most appropriate management beyond antibiotic therapy for this patient, given the strong likelihood of a peritonsillar abscess, would be (A) incision and drainage to directly address the core problem.

**AI_C:** Restating the Query: A young woman is suffering from a recent sore throat, fever, voice changes, and painful swallowing. She has been trying to manage these symptoms with acetaminophen. There are no significant entries in her medical history, and she's not on any regular medication. Her vital signs indicate a high fever, tachycardia, normal respiratory rate, and borderline low blood pressure, but her oxygen saturation is normal. The examination reveals inflamed and swollen oropharynx, tongue displacement, and a white exudate on her right tonsil. There is trismus and enlarged, tender nodes present in her anterior neck region. The question is to determine the best course of action, in addition to antibiotic treatment, from among the listed options. Planning: Let's consider each of the assumptions. (A) the patient may have an abscess that needs incision and drainage, (B) the inflammation might be severe enough that systemic corticosteroid therapy could help, (C) this could be a fungal tonsillitis - requiring nystatin, (D) it could be just a severe tonsillitis which would benefit from supportive care like gargling salt water, or (E) it might be severe enough to require surgical removal of the tonsils. Clinical Reasoning: The patient's symptoms, the physical appearance of her oropharynx, and her enlarged cervical nodes suggest some form of severe oropharyngeal infection. Trismus suggests the involvement of muscles of mastication, usually as a result of inflammation or infection. This, along with other signs like the uvula displacement and palatal edema, suggest a deeper infection that may have resulted in a peritonsillar abscess (PTA). PTAs can cause soporific voice changes (referred to as "hot potato voice"), due to the compromised movement of the oropharyngeal muscles. This deeply ensconced infection wouldn't be adequately managed or discovered by mere gargling with salt water or antifungal oral solutions. Systemic steroids might help reduce inflammation, but they won't address the source of the problem. Tonsillectomies are usually not done in acute settings because of the risk of uncontrolled bleeding from inflamed tissues. Concluding: Given the findings and the clinical thought process, it seems probable that this patient has a peritonsillar abscess. Antibiotics are needed to treat the underlying infection, but the abscess also needs to be drained. The broader infection might be causing her fever and general discomfort, but the abscess is probably the source of her trismus and voice changes. Antibiotics alone might not treat an abscess effectively because of a likely poor penetration of the drug into the pus within the abscess. Response: Therefore, the most appropriate management, besides antibiotic therapy, would be incision and drainage (Option A).

**Facilitator** The consensus among the AI responses is that the 22-year-old woman's symptoms and physical examination findings strongly suggest a peritonsillar abscess (PTA), a complication of an untreated, severe bacterial throat infection. The symptoms include a severe sore throat, fever, change in voice (often termed "hot potato voice"), and increased pain while swallowing. Physical examination findings such as uvular deviation, edema of the surrounding tissue, and neck lymphadenopathy further support this diagnosis. While antibiotic therapy is essential to manage the underlying infection, it wouldn't directly deal with the abscess, which can potentially obstruct the airway or contribute to ongoing infection. PTA is essentially a 'walled off' infection, and this compartmentalization may prevent adequate penetration of antibiotics. Therefore, in addition to antibiotic therapy, the abscess needs to be addressed directly. The other management options provided, such as intravenous methylprednisolone therapy, oral nystatin therapy, salt water gargle, and tonsillectomy, are not considered the most appropriate in this case. Intravenous methylprednisolone therapy and salt water gargle might help alleviate symptoms but would not address the core issue. Oral nystatin therapy would be used for a fungal infection, which this is not. Tonsillectomy is a more drastic measure typically reserved for recurrent episodes of tonsillitis or obstructive sleep apnea and would not generally be done in an acute infectious setting. Therefore, the consensus is that the most appropriate management for this patient, in addition to antibiotic therapy, would be (A) Incision and drainage. This approach would directly address the abscess, helping to speed up recovery, reduce pain, and prevent complications.
